# Protected stent retriever thrombectomy prevents iatrogenic emboli in new vascular territories

**DOI:** 10.1007/s00234-015-1583-8

**Published:** 2015-08-29

**Authors:** Pascal P. Klinger-Gratz, Gerhard Schroth, Jan Gralla, Simon Jung, Christian Weisstanner, Rajeev K. Verma, Pasquale Mordasini, Frauke Kellner-Weldon, Kety Hsieh, Mirjam R. Heldner, Urs Fischer, Marcel Arnold, Heinrich P. Mattle, Marwan El-Koussy

**Affiliations:** Department of Diagnostic and Interventional Neuroradiology, Inselspital, Freiburgstrasse 10, Bern University Hospital and University of Bern, 3010 Bern, Switzerland; Department of Radiology, University of Basel, Basel, Switzerland; Department of Neurology, Inselspital, Bern University Hospital and University of Bern, Bern, Switzerland

**Keywords:** Stroke, Stent retriever, SWI, Collaterals, TICI

## Abstract

**Introduction:**

Diagnostic tools to show emboli reliably and protection techniques against embolization when employing stent retrievers are necessary to improve endovascular stroke therapy. The aim of the present study was to investigate iatrogenic emboli using susceptibility-weighted imaging (SWI) in an open series of patients who had been treated with stent retriever thrombectomy using emboli protection techniques.

**Methods:**

Patients with anterior circulation stroke examined with MRI before and after stent retriever thrombectomy were assessed for iatrogenic embolic events. Thrombectomy was performed in flow arrest and under aspiration using a balloon-mounted guiding catheter, a distal access catheter, or both.

**Results:**

In 13 of 57 patients (22.8 %) post-interventional SWI sequences detected 16 microemboli. Three of them were associated with small ischemic lesions on diffusion-weighted imaging (DWI). None of the microemboli were located in a new vascular territory, none showed clinical signs, and all 13 patients have been rated as Thrombolysis in Cerebral Infarction (TICI) 2b (*n* = 3) or 3 (*n* = 10). Retrospective reevaluation of the digital subtraction angiography (DSA) detected discrete flow stagnation nearby the iatrogenic microemboli in four patients with a positive persistent collateral sign in one.

**Conclusion:**

Our study demonstrates two things: First, SWI seems to be more sensitive to detect emboli than DWI and DSA and, second, proximal or distal protected stent retriever thrombectomy seems to prevent iatrogenic embolization into new vascular territories during retraction of the thrombus, but not downstream during mobilization of the thrombus. Both techniques should be investigated and refined further.

## Introduction

Mechanical thrombectomy provides higher recanalization rates than intravenous or intraarterial thrombolysis, but clinical outcome is highly variable even with successful revascularization [1–14]. Good clinical outcome after mechanical thrombectomy, using mainly or exclusively thrombus fragmenting techniques and instruments (Penumbra trial, Multi MERCI trial, IMS III, MR RESCUE, SYNTHESIS) [6, 7, 15–17], is inferior even compared to studies using local arterial thrombolysis (PROACT II, MELT) [9, 18] or protected stent retriever thrombectomy (Dávalos et al., STAR trial, ESCAPE trial, EXTEND-IA trial, SWIFT-Prime) [2, 3, 11, 12, 14]. This might be explained partially by iatrogenic embolization into new vascular territories detected angiographically in 8.6 % of all patients in the groundbreaking Multicenter Randomized Clinical Trial of Endovascular Treatment for Acute Ischemic Stroke in the Netherlands (MR CLEAN) study [1]. Thrombectomy devices may fragment clots and create distal emboli that potentially disrupt collateral blood supply to the penumbra and deteriorate clinical outcome [19]. Animal in vivo studies have shown that thrombectomy devices can disrupt clots in different ways and cause variable rates of embolic events (EEs) [20]. To date, diffusion-weighted imaging (DWI) [21] and digital subtraction angiography (DSA) [15, 16] are considered as the best techniques for detection of emboli related to endovascular procedures. In acute stroke, however, the initial DWI lesion may mask secondary treatment-related emboli and DSA cannot reliably differentiate between no reflow and new EE. Moreover, only a minority of thrombus fragments can be detected on the pre-interventional diagnostic DSA [22]. Therefore, DSA cannot differentiate between a pre-existing thrombus fragment and an iatrogenic EE downstream the primary site of occlusion. In addition, still active collaterals can inhibit the antegrade bloodflow downstream the recanalized big vessel, especially if flow and pressure are diminished by mechanically induced vasospasms or if the control DSA has been performed with the 8- or 9-French guiding catheter placed in the internal carotid artery. Therefore, stagnating flow must not indicate distal emboli. Considering that the Thrombolysis in Cerebral Infarction (TICI) classification is normally based on a final selective one-vessel DSA, even a reduced parenchymal blush can be caused by still active collaterals, feeding non-contrasted blood into the contrasted vascular territory.

Susceptibility-weighted imaging (SWI), a relatively new technique in the primary and follow-up MRI evaluation of patients with acute stroke [23, 24], could probably overcome these limitations through its ability to detect even small emboli. In a recent study, SWI detected the occluding thrombus in 95 % of patients with stroke of the anterior circulation, and the presence of primarily fragmented thrombi before initiation of thrombolytic therapy predicted worse outcome [22].

The aims of the present study were to investigate the frequency of iatrogenic EE following protected stent retriever thrombectomy using SWI and to compare the results with those obtained with DWI and DSA.

## Methods

### Patients and clinical data

Three hundred eighty-four consecutive acute ischemic stroke patients treated with stent retriever thrombectomy at our stroke unit, from January 2010 to December 2013, were screened. Of these, 57 patients had acute ischemic stroke of the anterior circulation and underwent pre- and post-interventional MRI including DWI and SWI and were included in this retrospective study. All patients had anterior circulation strokes due to tandem occlusions of the internal carotid artery (ICA) and middle cerebral artery (MCA), distal ICA occlusions, carotid T occlusions, isolated occlusions of the MCA (M1 or M2 segment), or multiple occlusions of the anterior circulation (MCA in combination with anterior cerebral artery (ACA)).

Clinical data were recorded prospectively in our stroke registry. Cerebral reperfusion was assessed at the end of the endovascular intervention, on biplane or 3D rotational angiography, according to the TICI grading system [25]. The occurrence of symptomatic (sICH) and asymptomatic (aICH) intracranial hemorrhage was recorded according to the PROACT II criteria [18]. Clinical outcome was assessed 3 months after the ictus with the modified Rankin Scale (mRS).

### Imaging and treatment

Standard stroke MRI protocol was performed, which included DWI, T2-weighted imaging, fluid-attenuated inversion recovery (FLAIR), TOF magnetic resonance angiogram (MRA), SWI, first-pass gadolinium-enhanced MRA of the cervical and intracranial arteries, and perfusion imaging. The scans were acquired with a 1.5- and 3-T MRI system (Magnetom Avanto and Magnetom Verio; Siemens, Erlangen, Germany).

For the 1.5-T system, the SWI parameters were as follows: repetition time (TR) 49 ms, echo time (TE) 40 ms, number of averages 1, field of view (FoV) read 230 mm, FoV phase 75.0 %, voxel size 0.9 × 0.7 × 1.8 mm, flip angle 15°, and acquisition time 2 min and 59 s. For the 3-T scanner, the parameters were as follows: TR 28 ms, TE 20 ms, number of averages 1, FoV read 230 mm, FoV phase 75.0 %, voxel size 0.9 × 0.9 × 2.0 mm, flip angle 15°, and acquisition time 2:59 min. The SWI and minimum intensity projection (mIP) images were generated automatically by the scanner software.

As described previously [2–4], stent retriever thrombectomy was performed using a biplane, high-resolution angiographic system (Axiom Artis zee; Siemens, Erlangen, Germany). To reduce the risk of embolic complications, retrieval of the stent was performed in flow arrest using a balloon-mounted guiding catheter with its tip as close as possible to the skull base, to prevent the ICA from collapsing during aspiration. In addition, in patients with anatomically difficult access, a high-volume distal access aspiration catheter was used, if the 8- or 9-Fg balloon guiding catheter had to be inflated more proximally.

### Imaging analysis

MRI and DSA were evaluated retrospectively by two experienced neuroradiologists and a radiologist in training. Pre-interventional MRI was analyzed for the presence of a susceptibility vessel sign, and thrombus length was measured on SWI as described previously [22, 24].

**Fig. 1:**
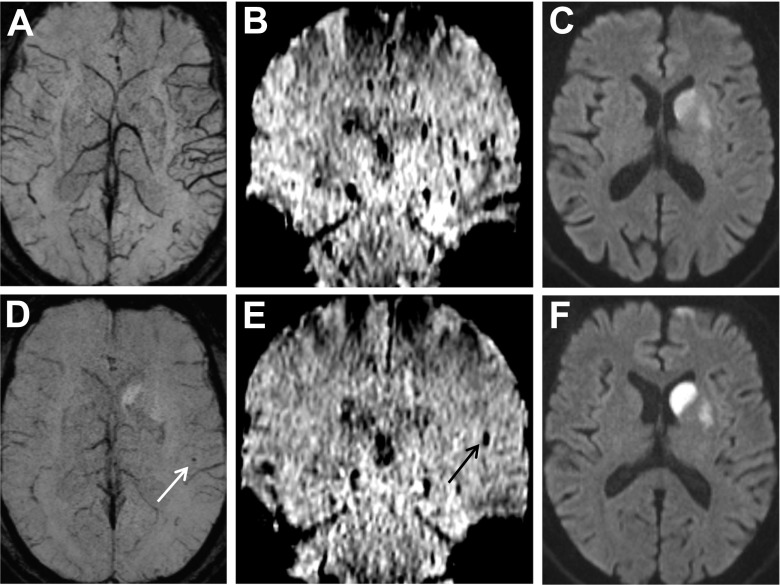
A 72-year-old man with mild aphasia and right-sided hemiparesis (NIHSS score 5). MRI shows left proximal MCA occlusion. On initial SWI, prominent veins in the region of the left Sylvian fissure are seen (**a** [axial, mIP] and **b** [coronal]). DWI shows diffusion restriction in the basal ganglia (**c**). Post-interventional SWI demonstrates a punctate signal drop corresponding to a peri-interventional embolus (**d** [*white arrow*, mIP] and **e** [*black arrow*]). The embolus is located outside of the infarcted area as seen on post-interventional DWI (**f**)

**Fig. 2:**
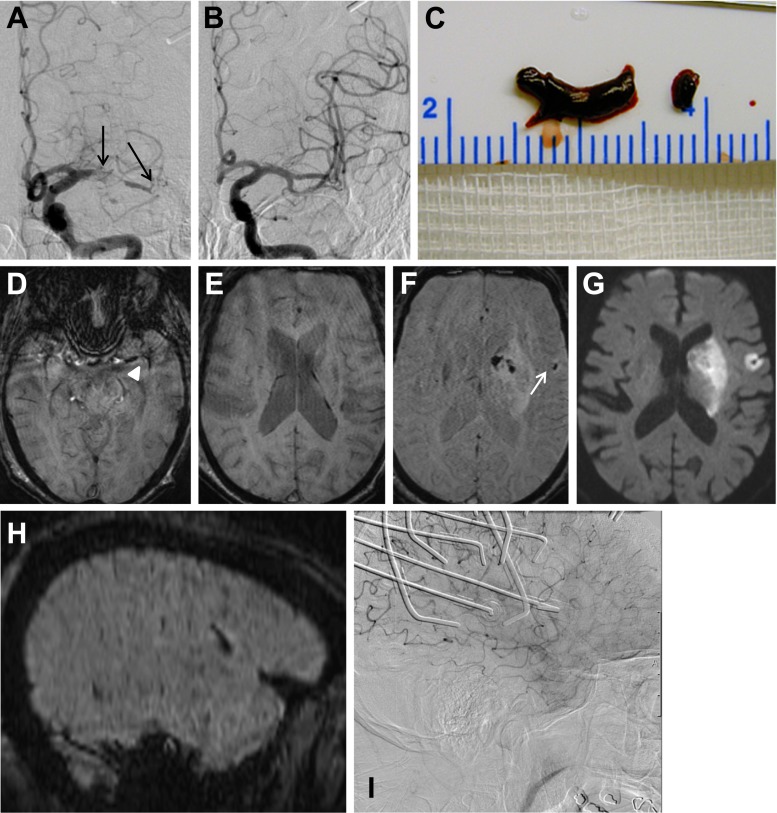
A 83-year-old man with right-sided hemiparesis (NIHSS score 21). Two separate thrombi in the left proximal M1 segment and the MCA bifurcation are seen on DSA (**a**, *black arrows*). Pre-interventional SWI shows thrombotic material in the proximal M1 segment and MCA bifurcation extending in an M2 branch (measuring 10 and 9 mm on SWI; **d**, *white arrowhead*) but no additional distal fragments (**e**). Two thrombus fragments were retrieved (**c**). Final biplane DSA control was rated as TICI 3 reperfusion (**b**). Post-interventional SWI detected one EE (**f**, *white arrow*) with an associated ischemic lesion on DWI (**g**). The sagittal reformatted projection of the SWI confirms the extracerebral location of the EE and its orientation along a suprasylvian MCA branch (**h**). Lateral DSA shows stagnating flow in the corresponding MCA branch with discretely diminished parenchymal blush during this phase of the DSA (**i**). (Artifacts are produced by simultaneous recording of NIRS)

**Fig. 3:**
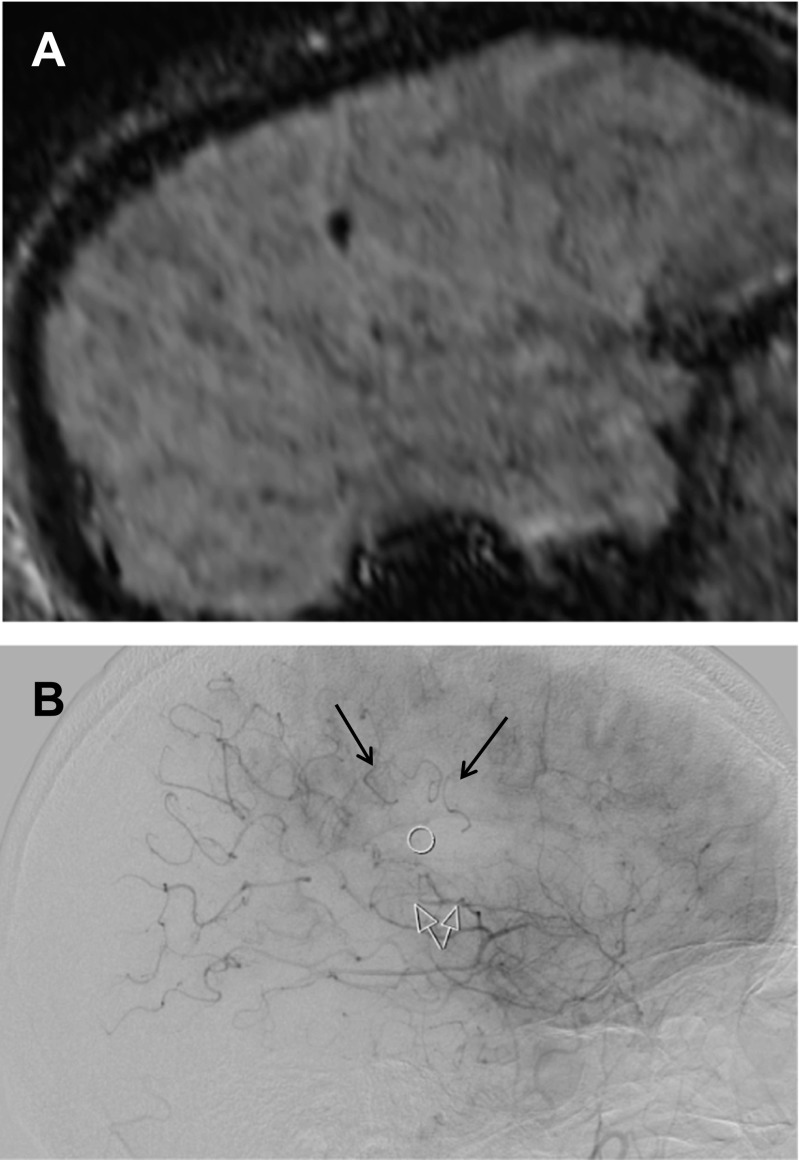
The post-interventional SWI of this 76-year-old man with a stroke of NIHSS 13 shows the EE in a parietal branch of the right MCA (**a**). As demonstrated by the frozen lateral DSA projection, proximal stagnating of contrast flow in the sylvian segment about 2 cm in front of the EE (*white ring*) is compensated by retrograde flow in the suprasylvian branches, which have been identified in the initial DSA as collaterals, coming down from the anterior cerebral artery (**b**, *black arrows*). The resulting minimal blush as well as the visualization of all arteries in the AP projection may have been the reason that the interventional team rated the final DSA series as TICI 3 in this patient. The stagnating flow in the distal temporo-occipital branches is not related to EE but to still active collaterals from the vertebrobasilar system

Post-interventional SWI was analyzed for signs of EE. To better discriminate emboli from vascular structures, additional minimum intensity (mIP, Fig. 1) and multiplanar (Figs. 2 and 3) projections of the SWI were reformatted. Emboli were defined as areas of tubular or dot-like signal drop not seen on pre-interventional SWI, following the direction of branches of the middle, anterior, or posterior cerebral arteries (Figs. 1, 2, and 3). Emboli were described as being located either inside or outside of the infarcted tissue, defined on post-interventional DWI and T2-weighted/FLAIR images.

**Fig. 4:**
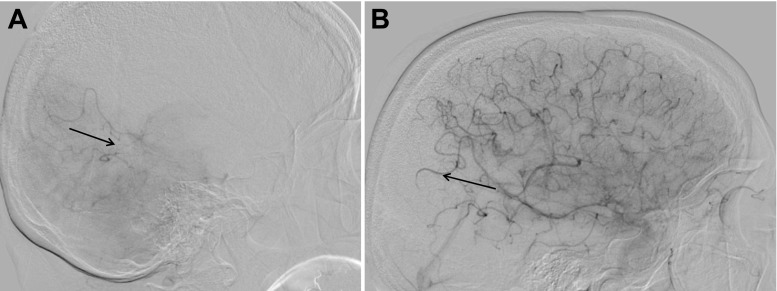
The DSA of the dominant vertebral artery in this 79-year-old woman with occlusion of the internal carotid artery shows the retrograde filling of the posterior parietal branches of the MCA (**a**). Due to the still active collaterals from the vertebrobasilar system, not visible in the selective DSA, the interventional team rated this as TICI 2b based on the stagnating flow and diminished temporooccipital blush (**b**). Quality control MR the next day found neither EE nor DWI lesions in this area

Biplane and/or 3D DSA was routinely performed after endovascular treatment and rated according to the TICI classification by the team of interventional neuroradiologists, who had performed the endovascular therapy (Table 1). When retrospectively reviewing the DWI and DSA, the raters were aware of the results of the SWI. For this reevaluation, the collaterals were identified in the pre-treatment DSA series, which are stored completely in our PACS system. Four- or at least three-vessel angiography (with opacification of the basilar artery via the dominant vertebral artery) is a mandatory part of our stroke protocol; thus, collaterals from the vertebrobasilar system (Fig. 4) and the contralateral side could be included in this evaluation. Re-examination of the post-interventional control DSA focused on the dynamic flow pattern of the arteries around the SWI-proven EE in at least two planes. The results were compared to the pre-treatment angioarchitecture, regarding if bloodflow of the collaterals was antegrade, stagnating, or still reversed and if the parenchymal blush was diminished. Control DSA was normally performed with the guiding catheter in the common carotid artery and the balloon fully deflated. Thus, disturbances of the flow could be avoided, and the flow pattern of the external carotid artery was used to calibrate the DSA as an internal quality control. Additional DSAs of the contralateral and posterior circulation have been performed frequently, if the watershed territory was not fully opacified in the selective ipsilateral DSA, and this information was also included in the TICI classification of the neuroradiologist in charge of the intervention (Table 1).

**Table 1:** Details of patients with emboli on post-interventional susceptibility-weighted imaging

Case	Age/sex	Occlusion site	Baseline NIHSS score	Stroke etiology	Reperfusion grade (TICI)	Number of emboli	Location of emboli (outside/inside infarct)	Emboli associated with new ischemia	mRS score at 3 months
1	67/M	ICA/M2	11	Large artery disease	3	1	Outside	No	3
2	62/M	M1	7	Cardioembolic	3	2	Outside	No	1
3	72/M	M1	5	Cardioembolic	3	1	Outside	No	0
4	48/F	M1	9	Unknown	3	1	Inside	No	0
5	70/F	M1	5	Cardioembolic	3	1	Outside	No	1
6	75/F	M1	21	Cardioembolic	3	1	Outside	Yes	4
7	38/F	M2	5	Other	3	1	Outside	No	1
8	73/F	M1	14	Unknown	3	1	Outside	No	Lost to follow-up
9	68/F	M1	23	Unknown	2b	1	Outside	No	0
10	77/M	M1	17	Large artery disease	2b	3	Inside	No	3
11	76/M	M2	13	Unknown	3	1	Inside	Yes	1
12	83/M	M1	21	Unknown	3	1	Outside	Yes	3
13	79/F	Distal ICA	14	Cardioembolic	2b	1	Inside	No	6

*NIHSS* NIH Stroke Scale, *TICI* Thrombolysis in Cerebral Infarction, *mRS* modified Rankin Scale, *M1* M1 segment of the MCA, *M2* M2 segment of the MCA

### Statistical analysis

Successful reperfusion was defined as TICI grades 2b–3. Clinical outcome was dichotomized into favorable (mRS scores 0–2) and poor outcome (mRS scores 3–6). The association of categorical variables with EE was analyzed with Fisher’s exact test. Continuous and ordinal variables were compared by Mann–Whitney *U* test. A probability value <0.05 was considered significant. Statistics were performed with SPSS Version 21 (IBM Corp., Armonk, NY).

## Results

### Occlusion characteristics and time point of imaging

Thirty-six patients had occlusions of the M1 segment, one of them in combination with a thrombus in the ipsilateral A3 segment. Five patients had isolated M2 occlusions. Nine patients presented with tandem occlusions affecting the ICA and ipsilateral MCA (M1 in eight patients, and M2 in one patient). In six patients, a carotid T occlusion was found. One patient had an occlusion of the distal ICA. Median NIH Stroke Scale (NIHSS) score on admission was 14 (range, 2–36).

Pre-interventional MRI was obtained 3.0 ± 2.3 h, and post-interventional MRI 43.6 ± 43.5 h after stroke onset. Median time from symptom onset or in case of wake-up stroke time last seen well, to initiation of endovascular intervention was 4.0 ± 2.4 h.

### Pre- and post-interventional imaging

Susceptibility vessel sign on pre-interventional SWI, corresponding to the proximal occluding thrombus, was detected in 56 of 57 patients (98.2 %). In one patient without susceptibility vessel sign, MRA showed an ICA but no MCA occlusion. Subsequent DSA demonstrated a tandem occlusion with thrombus located in the proximal and distal ICA and M1, most likely because of thrombus fragmentation and migration that occurred during intravenous bridging thrombolysis after the pre-interventional MRI and before start of the DSA.

Mean thrombus length, which could be measured on SWI in 49 patients, was 12.9 ± 7.1 mm. Fragmented thrombi on pre-treatment SWI were seen in one patient with an MCA thrombus and additional separate fragments in the M1 and M2 segments (Fig. 2).

### Imaging of new emboli and association with clinical parameters

New embolic lesions were found in 13 of 57 patients (22.8 %). Eleven patients had single emboli, one had two, and one three emboli (Table 1). All emboli were located in the MCA territory downstream to the occluding thrombus. No emboli were detected in the ACA and posterior cerebral artery territories. Eleven emboli occurred in patients with occlusions of the MCA, one with a tandem (ICA and M2), and one with a distal ICA occlusion. The emboli were located outside of the infarct core, defined by post-treatment DWI images, in nine patients and inside in four. Mean length of the distally migrated thrombus fragments on SWI was 5.2 ± 1.2 mm (range, 3.6–7.8 mm). Multiplanar reconstructions of the SWI images usually confirmed the ovoid shape of the EE, aligned to the course of the sylvian and suprasylvian branches of the MCA (Figs. 1, 2, and 3).

New ischemic lesions related to iatrogenic EE were detected on DWI in three patients (Figs. 2 and 3). Distal branch occlusions were visible on final post-thrombectomy biplane or 3D DSA in none of the patients with EE. Subsequently, recanalization had been rated as TICI 2b (*n* = 3) or TICI 3 (*n* = 10) in the initial radiological report. Reperfusion in the 44 patients without EE has been rated as TICI 0, *n* = 3; TICI 2a, *n* = 5; TICI 2b, *n* = 18; and TICI 3, *n* = 18.

Retrospective reevaluation, focusing on the area where the new EEs were localized on post-interventional SWI, detected stagnating flow in four patients, with two of them showing also new DWI lesions (Figs. 2 and 3). In one patient, persistent collateral sign was found, which is defined as persistent retrograde flow in arteries, which had been identified as collaterals on the pre-treatment DSA (Fig. 3).

EE occurred more often in patients treated under conscious sedation compared to general anesthesia (*n* = 8/20 (40.0 %) vs *n* = 5/37 (13.5 %); *P* = 0.044). EEs were less frequently observed with distal access catheter technique use compared to non-use (*n* = 1/15 (6.7 %) vs *n* = 12/42 (28.6 %)), but the difference was not significant (*P* = 0.150). Stroke etiologies were similar in both groups (*P* = 0.617) and so were thrombus length on SWI (*P* = 0.601) and duration from symptom onset to start of DSA (*P* = 0.488; Table 2).

**Table 2:** Anterior circulation stroke: clinical, procedural, and outcome parameters in patients with and without peri-interventional embolic events

	Patients with emboli (*n* = 13, 22.8 %)	Patients without emboli (*n* = 44, 77.2 %)
Intravenous thrombolysis, *n* (%)	6 (46.2)	21 (47.7)
Successful reperfusion (TICI 2b–3), *n* (%)	13 (100)	36 (81.8)
Interventional characteristics, *n* (%)		
Distal access catheter use	1 (7.7)	14 (31.8)
Multimodal therapy	5 (38.5)	23 (52.3)
Thromboaspiration	3 (23.1)	15 (34.1)
Extracranial and intracranial stenting	1 (7.7)	9 (20.5)
Intraarterial thrombolysis	1 (7.7)	9 (20.5)
Anesthesia, *n* (%)		
General anesthesia	5 (38.5)	32 (72.7)
Conscious sedation	8 (61.5)	12 (27.3)
Symptomatic ICH, *n* (%)	0 (0.0)	3 (6.8)
Asymptomatic ICH, *n* (%)	3 (23.1)	8 (18.2)
Favorable outcome (mRS score 0–2) at 3 months, *n* (%)	7/12 (58.3)	29/41 (70.7)
Dead at 3 months, *n* (%)	1/12 (8.3)	3/43 (7.0)
Stroke etiology, *n* (%)		
Large artery disease	2 (15.4)	8 (18.2)
Cardioembolic	5 (38.5)	20 (45.5)
Other determined etiology	1 (7.7)	4 (9.1)
Unknown aetiology	5 (38.5)	12 (27.3)
Atrial fibrillation, *n* (%)	5/12 (41.7)	16/39 (41.0)
Thrombus length on pre-interventional SWI (mm), mean (SD)	13.4 (10.1) (*n* = 12)	12.8 (6.1) (*n* = 37)

*TICI* Thrombolysis in Cerebral Infarction, *mRS* modified Rankin Scale, *ICH* intracranial hemorrhage

In patients with a clinical follow-up at 3 months, favorable clinical outcome (mRS score 0–2) was achieved in 7 of 12 patients (58.3 %) and 29 of 41 patients (70.7 %) with and without EE, respectively (*P* = 0.490). One of 12 patients (8.3 %) with and 3 of 43 patients (7.0 %) without emboli died within 3 months (*P* = 1.000).

## Discussion

Clinical outcome after mechanical thrombectomy is highly variable despite high revascularization rates [1–12]. Clinical outcome, among other factors, is device-dependent, with stent retrievers showing higher rates of recanalization and favorable outcome than devices that cause thrombus fragmentation [5, 10]. This was confirmed by the SWIFT and TREVO 2 studies [5, 10]; however, for both MERCI and the stent retriever groups, the rate of good clinical outcome was lower than in old studies on intraarterial thrombolysis, despite that NIHSS is comparable and treatment selection was less restrictive in PROACT 2, MELT [9, 18], and the Swiss study [26], comparing intravenous (iv) and intraarterial (ia) thrombolysis (Table 3). The risk of embolic complications is of particular concern in mechanical thrombectomy, as emboli can impair collateral blood supply to the affected territory, accelerate penumbral tissue loss, and cause additional ischemic lesions [19]. This is in accordance with a study demonstrating that the presence of multiple thrombi before initiation of thrombolytic therapy predicts worse outcome [22].

**Table 3:** Trials on endovascular treatment of anterior circulation stroke: clinical outcome in relation to baseline NIHSS score and endovascular techniques applied

Study designation	Number of patients undergoing endovascular treatment	Baseline NIHSS score, median	Favorable outcome (mRS score 0–2) at 3 months (%)	Endovascular techniques applied
PROACT II [18]	121	17	40	ia prourokinase
MELT [9]	57	14	49	ia urokinase
Mattle et al. [26]	55	17^a^	53	ia urokinase
Galimanis et al. [31]	623	15	49	ia urokinase, aspiration, stent retriever (Solitaire)
Multi MERCI trial [7]	164	19	36	Distal Thrombectomy (Merci)
Penumbra trial [6]	125	18^a^	25	Fragmentation/aspiration (Penumbra)
SWIFT [10]				
- Merci	58	18	28	Merci
- Solitaire	55	17	37	Solitaire (protection not mandatory)
TREVO 2 [5]				
- Merci	90	18	22	Merci
- TREVO	88	19	40	Stent retriever (Trevo, protection not mandatory)
MR CLEAN [1]	233	17	33	ia alteplase or urokinase, thrombus retraction, aspiration, wire disruption, stent retriever (protection not mandatory)
IMS III [15]	434	17	41	Merci, Penumbra, Solitaire (protection not mandatory), ia tPA
MR RESCUE [16]	64	16 (penumbral), 19 (non-penumbral	19	Merci, Penumbra, ia tPA
SYNTHESIS [17]	181	13	42	ia tPA, wire disruption, Merci, Penumbra, Trevo, Solitaire (protection not mandatory)
Dávalos et al. [2]	141	18	55	Solitaire (protection in 74 % of interventions)
STAR trial [3]	202	17	58	Solitaire (protection mandatory)
ESCAPE trial [11]	165	16	53	Stent retriever recommended (protection recommended)
EXTEND-IA trial [12]	35	17	71	Solitaire (protection mandatory)
REVASCAT [13]	103	17	44	Solitaire (protection not mandatory)
SWIFT prime [14]	98	17	60	Solitaire (protection mandatory)

*ia* intraarterial, *NIHSS* NIH Stroke Scale, *mRS* modified Rankin Scale

^a^Mean

In the present study, we analyzed a series of patients undergoing protected stent retriever thrombectomy. Mechanical thrombectomy was performed in flow arrest and under aspiration using a balloon-mounted guiding catheter, a distal access catheter, or both. Peri-interventional EEs following thrombectomy were detected on SWI in 22.8 % of patients. Emboli were mostly solitary and showed a similar signal intensity as the primary occluding thrombus. Emboli were exclusively located in the vascular territory distal to the primary arterial occlusion and therefore resulted most likely from fragmentation during thrombus mobilization.

On DWI, new ischemic lesions associated with EE were found in three of 16 EEs. Even in retrospect, stagnation or reversed flow in the vicinity of the EE in addition with a subtle decrease in parenchymal blush could be presumed in four patients only (Figs. 2 and 3). These subtle signs of local flow restriction were visible when the images of the lateral projection of the DSA were evaluated dynamically, but not on the AP view or in the 3D rotation angiography.

The small size of the EE may be one reason for the restricted visibility in the DSA. In contrast to EEs in new vascular territories, which are normally well visible even on frozen DSA images (1–3), downstream EEs migrate only to the watershed zone, where the antegrade flow and pressure are in balance with the reversed flow of the collaterals. Therefore, comparable with embolic basilar artery occlusion, downstream iatrogenic EEs are not fixed and compressed in an artery with a diameter smaller than the EE, which can result in small oscillations of the thrombus and discrete bloodflow around the EE. This may be another reason, why it is difficult to detect downstream EE as a clear-cut branch occlusion. Considering the sensitivity of DSA to motion artifacts, it is not a surprise that indirect discrete signs of flow stagnation may be obliterated and may be detected retrospectively only, after post-processing of the DSA, including motion correction.

Currently, modified TICI is considered the optimal grading system for the evaluation of endovascular recanalization [27]. In view of our finding that the majority of peri-procedural EEs are invisible in DSA, post-treatment SWI may be a valuable complement to DSA for assessment of the success and safety of endovascular procedures.

Peri-interventional emboli, as detected on DSA, were found in up to 48 % of patients treated with the Penumbra system [28], and an analysis of the histological structure of clots extracted by the Merci retrieval system reported that in 64 %, thrombi were retrieved in multiple fragments [29]. In the MR CLEAN study using various types of intraarterial techniques, iatrogenic embolization to primarily non-affected vascular territories was observed in 8.6 % of patients and 5.6 % had clinical signs of a new ischemic stroke [1]. A blinded core lab found nine new emboli (6 %), two of them in primarily non-occluded arteries (1.4 %), in a multicenter study, using the Solitaire stent retriever with balloon protection in 74 % of interventions [2]. In a study with mandatory use of balloon protection, two emboli were detected in 202 patients (1 %) by the corelab and no embolic event was observed in the subgroup of 119 patients treated with iv thrombolysis in addition to protected stent retriever thrombectomy [3]. The low rate of embolization to primarily non-affected vascular territories, serving as collaterals to the penumbral tissue [19], might contribute to the better clinical outcomes seen in the latter studies [2, 3] compared to those using predominantly devices and techniques that achieve recanalization through thrombus fragmentation [6, 7, 15–17] or stent retriever thrombectomy without mandatory protection [1, 5, 10] (Table 3).

Modern CT [30] and MRI techniques [24] allow pre-interventional visualization of thrombus length and shape, helping the interventionist to deploy the stent retriever over the whole thrombus length. In our study, retraction of the thrombus was performed in flow arrest and simultaneous aspiration using a balloon-mounted guiding catheter. In addition, in patients with anatomically difficult access, a distal access catheter was used. This might explain the fact that distal emboli were only detected in the vascular territory distal to the primary occlusion and not in adjacent territories (e.g., ACA territory). The tiny EEs seen downstream to the primary occlusion site may have been caused by shearing-off of small thrombus parts to perforating arteries during thrombus mobilization, a phenomenon that could be observed in animal models using radiopaque thrombi [20]. Creation of the iatrogenic EE during the passage of the occlusion is unlikely, because we know that the microcatheter never penetrates the thrombus itself but passes the site of occlusion between the vessel wall and the thrombus [31]. In addition, careful microcatheter contrast injection distal to the occlusion is a mandatory part of our stroke protocol, to confirm that the size of the artery is suitable to deploy the stent retriever. However, we have never been able to detect distal EE, neither primary fragmented thrombi, as discussed recently in a theoretical framework [21], nor iatrogenic EE.

Interestingly, this assumption may be supported by a trend for less frequent EE when distal access catheters were used (6.7 vs 28.6 %), but the difference was not significant (*P* = 0.150). Distal access catheters may facilitate the mobilization of thrombi by adding the suction power of the aspiration catheter to the traction force of the stent retriever, reducing the risk of distal embolization during thrombus mobilization. In addition, the distance the thrombus has to be retracted through the vessel is minimized, thus protecting the origins of arterial branches that have to be passed during retraction.

The rates of favorable clinical outcome at 3 months were not significantly different in patients with and without emboli (58.3 vs 70.7 %; *P* = 0.490). This seems to be in contrast to a recent study that demonstrated that primarily fragmented thrombi detected before initiation of thrombolytic therapy predicted worse outcome [22]. This discrepancy could have three reasons. First is that the number of patients with EE was too small to show a significant difference. Second is that emboli seen in our patient cohort were smaller than those in patients with multiple thrombi before initiation of thrombolytic therapy [22]. In fact, only a minority of patients with EE showed signs of tissue damage on DWI that could be attributed to iatrogenic emboli. Last, the reason for the good clinical outcome might be that no embolization to primarily non-affected vascular territories occurred in patients with and without EE.

EEs have been found in a significantly higher percentage of patients who underwent thrombectomy with conscious sedation compared to patients treated with general anesthesia (40.0 vs 13.5 %; *P* = 0.044). Unlike in general anesthesia, patients with conscious sedation often move during the potentially painful mobilization and retraction of the thrombus. Patient movement might increase the risk of embolization, as it impairs the quality of flow arrest and aspiration and mobilization and retraction of the thrombus cannot be performed in a slow and controlled fashion.

Our study has some limitations. First, not all EEs seen on post-interventional SWI may have actually been related to mechanical thrombectomy itself. New EEs could also be related to ongoing embolization from the primary embolic source of the stroke or migration of thrombus remnants still in place after thrombectomy. Second, the extensive artifacts on SWI generated in the proximity of the skull base limit the evaluation of the adjacent brain parenchyma, the vessels of the posterior circulation, and the intracranial parts of the ICA. Third, the distinction between embolus and hemorrhagic transformation on SWI can be equivocal if the hypointensities are located inside infarcted parenchyma. In such patients, only isolated dot-like lesions were counted as emboli. However, the majority of EEs were detected outside the infarcted territory (*n* = 9/13), making hemorrhagic transformation very unlikely. Moreover, most EEs were of a slightly elongated shape (Figs. 1, 2, and 3), due to the orientation of the thrombotic material in the vessels. This helps to distinguish them from cerebral microbleeds, usually presenting spherical in SWI [32]. Quantitative susceptibility mapping (QSM) was performed in a few of our patients only but may improve the specificity of this new source of MR image contrast [33, 34].

Fourth, in many patients, the time span between endovascular treatment and post-treatment imaging was quite long. Mean size of the EE as measured on SWI was 5.2 mm. However, the length of the thrombi may be shorter due to the blooming effect [24] and oscillations of the downstream EE, as described above. It cannot be excluded that ongoing thrombolysis may have caused the disappearance of some peri-interventional EEs on post-treatment SWI. However, EEs were equally distributed in patients treated primarily by endovascular approach (23 %; seven EEs in 30 patients) and the bridging group (22 %; six EEs in 27 patients), but the number of patients is too small, to draw any conclusion.

Fifth, the field strength of the MRI scanners was not standardized across patients because we use several scanners in clinical stroke imaging. Previous studies have shown that the detectability of cerebral microbleeds on SWI improves with increasing field strength [35]. Thus, the detection rate of EE is most likely influenced by field strength as well. Nonetheless, this phenomenon did not seem to have a major impact on our study results, as six EEs (46.2 %) were detected on SWI acquired with a 1.5-T and seven EEs (53.8 %) with a 3-T MRI.

## Conclusions

This study demonstrates that SWI can be applied to detect peri-interventional microemboli that are only partially seen on follow-up DWI or DSA, currently the standard techniques used for the detection of EE related to endovascular procedures [1, 15, 16, 21]. The possibility to non-invasively visualize iatrogenic EE makes SWI an interesting tool to evaluate the quality of thrombectomy techniques and to improve instruments and techniques for endovascular stroke treatment. This quality control is needed to evaluate the safety of thrombectomy devices and techniques before they are used in patients for clinical routine as well as in clinical trials. SWI before and after endovascular, image-guided, microneurosurgical stroke therapy confirms that stent retriever combined with emboli protection techniques may prevent iatrogenic embolization to primarily non-affected vascular territories during the retraction of the thrombus, however, not downstream emboli during its mobilization.
